# Development of an Infectious Cell Culture System for Hepatitis C Virus Genotype 6a Clinical Isolate Using a Novel Strategy and Its Sensitivity to Direct-Acting Antivirals

**DOI:** 10.3389/fmicb.2018.02950

**Published:** 2018-12-04

**Authors:** Mingxiao Chen, Fuxiang Zheng, Guosheng Yuan, Xiaobing Duan, Liang Rong, Junwei Liu, Shengjun Feng, Ziting Wang, Min Wang, Yetong Feng, Qing Zhou, Jinqian Li, Kai Deng, Chunna Li, Jinyu Xia, Guirong Rao, Yuanping Zhou, Yongshui Fu, Yi-Ping Li

**Affiliations:** ^1^Institute of Human Virology and Zhongshan School of Medicine, Sun Yat-sen University, Guangzhou, China; ^2^Key Laboratory of Tropical Disease Control of Ministry of Education, Sun Yat-sen University, Guangzhou, China; ^3^Department of Infectious Diseases, Nanfang Hospital, Southern Medical University, Guangzhou, China; ^4^Guangzhou Blood Center, Guangzhou, China; ^5^Program of Pathobiology, The Fifth Affiliated Hospital and Zhongshan School of Medicine, Sun Yat-sen University, Guangdong, China; ^6^Key Laboratory of Liver Disease, Center of Infectious Diseases, PLA 458 Hospital, Guangzhou, China

**Keywords:** hepatitis C virus, genotype, cell culture system, adaptive mutation, consensus sequence, direct-acting antiviral agents

## Abstract

Hepatitis C virus (HCV) is classified into seven major genotypes, and genotype 6 is commonly prevalent in Asia, thus reverse genetic system representing genotype 6 isolates in prevalence is required. Here, we developed an infectious clone for a Chinese HCV 6a isolate (CH6a) using a novel strategy. We determined CH6a consensus sequence from patient serum and assembled a CH6a full-length (CH6aFL) cDNA using overlapped PCR product-derived clones that shared the highest homology with the consensus. CH6aFL was non-infectious in hepatoma Huh7.5 cells. Next, we constructed recombinants containing Core-NS5A or 5′UTR-NS5A from CH6a and the remaining sequences from JFH1 (genotype 2a), and both were engineered with 7 mutations identified previously. However, they replicated inefficiently without virus spread in Huh7.5 cells. Addition of adaptive mutations from CH6a Core-NS2 recombinant, with JFH1 5′UTR and NS3-3′UTR, enhanced the viability of Core-NS5A recombinant and acquired replication-enhancing mutations. Combination of 22 mutations in CH6a recombinant with JFH1 5′UTR and 3′UTR (CH6aORF) enabled virus replication and recovered additional four mutations. Adding these four mutations, we generated two efficient recombinants containing 26 mutations (26m), CH6aORF_26m and CH6aFL_26m (designated “CH6acc”), releasing HCV of 10^4.3^–10^4.5^ focus-forming units (FFU)/ml in Huh7.5.1-VISI-mCherry and Huh7.5 cells. Seven newly identified mutations were important for HCV replication, assembly, and release. The CH6aORF_26m virus was inhibited in a dose- and genotype-dependent manner by direct-acting-antivirals targeting NS3/4A, NS5A, and NS5B. The CH6acc enriches the toolbox of HCV culture systems, and the strategy and mutations applied here will facilitate the culture development of other HCV isolates and related viruses.

## Introduction

Infection with hepatitis C virus (HCV) is a major cause of chronic hepatitis, which could progress to liver cirrhosis and liver cancer. Treatment of hepatitis C has achieved great success after gradually spreading use of direct-acting antiviral agents (DAAs) (Pawlotsky et al., 2015). Approximately 71 million of world population are chronically infected with HCV, and ∼390,000 deaths annually are related to HCV. It is still a challenge to increase the accessibility of DAAs for more hepatitis C patients in need, and importantly there is no vaccine available for HCV infection. Thus, HCV remains a health threat for the world population.

HCV is classified into genus *Hepacivirus* of the *Flaviviridae* family. The virus genome is a positive-sense single-stranded RNA of ∼9600 nucleotides (nts) containing one open reading frame (ORF) and untranslated regions (UTRs) at 5′ and 3′ ends (5′UTR and 3′UTR). The ORF encodes a polyprotein of ∼3011 amino acids (aa), which is cleaved into 10 viral proteins, including structural proteins Core, E1, and E2 and non-structural proteins p7, NS2, NS3, NS4A, NS4B, NS5A, NS5B (Scheel and Rice, 2013).

HCV genome is highly divergent and has been classified into 7 major genotypes and more than 67 subtypes based on its sequence difference (Smith et al., 2014). Major genotypes, subtypes, and isolates differ from each other by ∼30, ∼20, and ∼2–10%, respectively, at the nucleotide and amino acid levels (Bukh et al., 1993; Smith et al., 2014). Sequence differences are associated with clinical outcome and sensitivity to neutralizing antibodies and therapy (Meunier et al., 2005; Gottwein et al., 2009; Bukh, 2016).

Prevalence of HCV genotypes varies, and global surveys show that genotypes 1, 2, 3, 4, 5, and 6 account for 46, 9, 30, 8, 1, and 6% of all infections, respectively (Messina et al., 2015). HCV genotypes distribute geographically (Mohd Hanafiah et al., 2013), and genotype 6 is common and increasing in East- and Southeast Asian countries, responsible for 6–20% of all HCV infections in southern China and Vietnam (Li C. et al., 2014; Chen et al., 2017) and up to 95% in Laos (Hubschen et al., 2011; Gower et al., 2014). Genotype 6 is found to have the greatest genetic diversity, of which at least 24 subtypes (6a-6xa) have been identified (Zhang et al., 2017). Besides, accumulating evidences show that genotype 6a is closely associated with intravenous drug use (Simmonds, 2013) and may also increase the risk of developing liver cancer among Asian patients with cirrhosis (Lee et al., 2017). Thus, more efforts are urgently needed to study the HCV genotype 6.

Efficient HCV cell culture systems are essential for the studies of various aspects of the HCV life cycle, pathogenesis, development of vaccine and antivirals. However, inability to directly culture HCV *in vitro* has limited our understanding of HCV and its related diseases. JFH1 (genotype 2a) clone is still the unique strain that is able to replicate in hepatoma Huh7 cells and derivatives without requirement of adaptive mutations (Wakita et al., 2005; Zhong et al., 2005), however, addition of adaptive mutations improves the infectivity (Delgrange et al., 2007; Russell et al., 2008). Using the replication capacity of JFH1, we and others have developed various HCV chimera recombinants containing different sequence regions of genotypes 1–6 (Lindenbach et al., 2005; Pietschmann et al., 2006; Gottwein et al., 2007, 2009; Jensen et al., 2008; Scheel et al., 2008; Li et al., 2011b; Li Y.P. et al., 2014). Recently, culture infectious full-length HCV clones of genotypes 1a, 2a, 2b, and 3a independent of JFH1 were developed by introduction of adaptive mutations (Date et al., 2012; Li et al., 2012a,b; Lu et al., 2014; Ramirez et al., 2014; Li Y.P. et al., 2014; Ramirez et al., 2016). Two genotype 6a clones for the viruses isolated in 1990’s were recently developed using similar approaches (Kolykhalov et al., 1996, 1997; Boson et al., 2011; Pham et al., 2018), during the preparation of this manuscript. All these recombinants require adaptive mutations for replication initiation and virus production, thus identification of such adaptive mutations is important and applicable for culture development of other clinical isolates.

In this study, we developed an infectious full-length HCV genotype 6a clone for a Chinese clinical isolate and designated it CH6acc. Using a strategy that combined the adaptive mutations and a cDNA clone shearing high homology with the consensus sequence, we demonstrated that the CH6acc replicated efficiently in hepatoma Huh7.5 cells and mCherry-reporter Huh7.5.1 cells The functional roles of newly identified mutations and the sensitivity to DAA treatment were also explored.

## Materials and Methods

### Ethics Statement

The use of patient serum was approved by the Medical Ethics Committee at the Zhongshan School of Medicine, Sun Yat-sen University (No. 2014-072). HCV infected sera were collected and anonymized by the Fifth Affiliated Hospital of Sun Yat-sen University, the Nanfang Hospital of Southern Medical University, the PLA 458 Hospital, and the Guangzhou Blood Center, Guangzhou, China.

### HCV Infected Serum and Plasmids

The HCV RNA level in the patient serum was determined by the HCV Nucleic Acid Detection Kit (TaqMan) (Daangene, China). From serum-extracted RNA, five reverse transcription-PCR (RT-PCR) amplicons overlapping to cover the nearly complete genome from 5′UTR to 3′UTR X-tail (nucleotides 60-9570) were TA-cloned. The primers used for RT-PCRs of both patient serum and cultured viruses are listed in Supplementary Table S1. Six to twelve clones of each amplicon were sequenced to deduce a consensus sequence. The fragments were selected and assembled to the backbone plasmids (below) using standard cloning procedures or chemically synthesized the fragments. Mutations were introduced by procedures including site-directed mutagenesis, fusion PCR, or chemical synthesis (Synbio Technologies, China). The plasmids pJ6^5′UTR-NS2^/JFH1 (Li et al., 2011a), pHK6a^5′UTR-NS5A^/JFH1 (Li Y.P. et al., 2014), and pJ6/JFH1-NS5AΔ40-EGFP (Gottwein et al., 2011a) were used as the backbone to construct CH6a Core-NS2, 5′UTR-NS5A or Core-NS5A, CH6a ORF and full-length recombinants, respectively. Final plasmid preparations were confirmed by Sanger sequencing analysis.

### Transfection and Infection of HCV in Cultured Cells

The human hepatoma cell line Huh7.5, generously provided by Dr. Charlies M. Rice (Apath, L.L.C and Rockefeller University), was maintained in Dulbecco’s modified Eagle medium (DMEM) (Life Technologies) supplemented with 10% fetal bovine serum (FBS) (Hyclone, United States), 100 U/ml of penicillin and 100 μg/ml of streptomycin (Life Technologies) at 37°C with 5% CO_2_. Huh7.5.1 cells containing an HCV infection-activated split-intein-mediated reporter system (VISI), (Huh7.5.1-VISI-mCherry cells) were generously provided by Dr. Gang Long (Institut Pasteur of Shanghai, Chinese Academy of Sciences) and maintained as reported (Zhao et al., 2017). Huh7.5.1 cells were provided by Dr. Francis V. Chisari (Scripps Research Institute, United States) (Zhong et al., 2005). Cells were seeded in 6-well plates (∼4.0 × 10^5^ cells/well) and allowed to grow to 80% confluence at the time of transfection and infection. Transfection and infection procedures were previously described (Li et al., 2012b). The transfected or infected cultures were incubated for ∼16 h, and the cells were split to sub-cultures every 2 days.

To monitor HCV infection in the transfected and infected cultures, immunostaining using monoclonal anti-HCV Core antibody C7-50 (Santa Cruz Biotechnology, United States) or directly visualization of mCherry expression of Huh7.5.1-VISI-mCherry cells using fluorescence microscope were performed as previously described (Li et al., 2012a; Zhao et al., 2017). Percentage of HCV-positive cells in the culture was estimated as spread kinetics of HCV in the cultures. When 80% of cells were HCV antigen positive (peak infection), culture supernatants were collected, filtered (0.45 μm), and stored at -80°C for future analysis. The sequences of HCV recombinants were determined by a long RT reaction, 2–4 overlapping PCRs covering entire ORF and partial 5′UTR and 3′UTR, and direct sequencing analysis of PCR products.

### Focus Forming Unit (FFU) Assay

Hepatitis C virus infectivity titers in the supernatant were determined by focus forming unit (FFU) assay as previously described (Li et al., 2011a). Briefly, 6 × 10^3^ Huh7.5 cells per well were seeded in 96-well plates and grew for 24 h. The diluted HCV-containing culture supernatant was added and incubated for 48 h. Then, the HCV infected cells were fixed with methanol (-20°C), immunostained with anti-HCV Core antibody C7-50 in 1/500 dilutions and visualized with secondary antibody Alexa Fluor^®^488 Goat Anti-Mouse IgG (H+L) or Alexa Fluor^®^594 Goat Anti-Mouse IgG (H+L) (Life Technologies) in 1/500 dilutions. The number of FFU was manually counted using fluorescence microscopy (Leica Microsystems).

### Determination of Intracellular and Extracellular HCV Infectivity Titers and Core Levels

For single-cycle production assays, HCV RNA was transfected into a Huh7-derived CD81-deficient cell line, S29 (Russell et al., 2008). Intracellular and extracellular HCV infectivity titers, as well as HCV Core levels, were determined 48 h post transfection. Briefly, S29 cells of 3.5 × 10^5^ cells/well were seeded in 6-well plates 24 h prior to transfection. HCV RNA of 5 μg was transfected, and at 48 h post transfection culture supernatants were collected, filtered, and used for determination of extracellular Core levels by Western blotting and of infectivity titers by FFU assay. For intracellular infectivity titers, transfected S29 cells were harvested and washed three times with PBS and then resuspended in complete DMEM and subjected to five quick freeze-thaw cycles to release intracellular virus particles. Cell debris was removed by centrifugation at 14,000 rpm for 15 min. HCV infectivity titers were performed in triplicate determinations, and FFU were enumerated by manual count. Both intracellular and extracellular Core antigen levels were determined by mouse anti-HCV Core antibody C7-50. The secondary antibody was Goat anti-mouse IgG(H+L)-horseradish peroxidase (HRP) (Ruikang, China). The β-actin was determined by HRP-anti-β-actin mouse monoclonal antibody (Proteintech, China).

### Quantitative Detection of HCV RNA

HCV RNAs were isolated from S29 cells and culture supernatants using TRIzol/chloroform extraction procedures (Life Technologies). For reverse transcription (RT), 500 ng of RNA was used by following the manufacture’s protocol of the HiScript II Q RT SuperMix kit (Vazyme, China), which included thermal reactions of 42°C for 2 min, 55°C for 15 min and 85°C for 5 s. One microliter of cDNA (1:10 dilution) was applied to quantitative PCR (qPCR) by using a StepOne qRT-PCR SYBR Green PCR Kit (Vazyme). HCV-specific qPCR was conducted in triplicate using a FastStart Universal SYBR Green Master (ROX) (Vazyme). The HCV-specific primers used in quantification was the following: HCV qS: 5′- CTTCACGCAGAAAGCGCCTA- 3′ and HCV qAS: 5′-CAAGCGCCCTATCAGGCAGT-3′ (Boson et al., 2011). Reactions were performed by one cycle of 95°C for 5 min, followed by 40 thermal cycles consisting of 95°C for 15 s and 60°C for 1 min. The amount of HCV RNA was calculated by a standard curve made from a serial dilution of a full-length HCV genomic plasmid, of which the copy number of DNA molecules was quantitated.

### Western Blotting

HCV Core levels in transfected Huh7.5 and S29 cells, as well as in culture supernatant, were determined by Western blotting. Briefly, for intracellular Core, HCV RNA-transfected or virus-infected cells were lysed use 1× lysis buffer [6× lysis buffer: 300 mM Tris-HCl (pH = 6.8), 12% sodium dodecyl sulfate (SDS), 60% glycerol, 0.6% bromophenol blue], and then cell lysates were subjected to protein denaturation at 98°C for 8 min. Samples were separated through the precasted 12% SDS-polyacrylamide gels. Afterwards, the separated proteins were transferred to a Hybond^®^-P polyvinylidene difluoride (PVDF) membrane (Bio-Rad, United States), and then membranes were washed with PBS containing 1% Tween-20 (PBS-T) and blocked with 5% skim milk. The blocked membranes were incubated overnight at 4°C with anti-HCV Core antibody C7-50 or HRP-anti-β-actin with gentle rotation. After washing with PBS-T, immunoblotting was done by incubation (1 h) with the enhanced chemiluminescence (ECL) sheep anti-mouse IgG HRP-linked whole antibody (Ray Antibody Biotech, China).

### Treatment of Direct-Acting Antiviral Agents (DAAs) for Different HCV Genotype Viruses

Direct-acting antiviral agents were purchased (MedChemExpress, China) and dissolved in dimethyl sulfoxide (Sigma-Aldrich, China). Treatment experiments were performed as described previously (Li et al., 2012a; Li Y.P. et al., 2014), with minor modifications. Briefly, Huh7.5 cells (6,000 cells/well) seeded in 96-well plates were infected with HCV recombinant viruses in triplicate for 24 h, and then treated with DAAs in the concentrations that did not cause cytotoxicity (determined in pilot experiments). Alternatively, Huh7.5 cells with HCV peak infection (80% cultured cells positive for HCV) were treated with DAAs. After a 48-h treatment, cells were fixed with methanol (-20°C), and HCV Core positive cells were determined by immunostaining with anti-HCV Core C7-50 antibody and ECL TM^®^ anti-rabbit IgG-HRP-linked whole antibody (GE Healthcare, United Kingdom) and visualized by DAB Substrate Kit (Abcam, United Kingdom). The dose-response curves of the viruses were plotted using GraphPad Prism 5.0 software.

## Results

### Genome Sequence of CH6a Isolate

**FIGURE 1:**
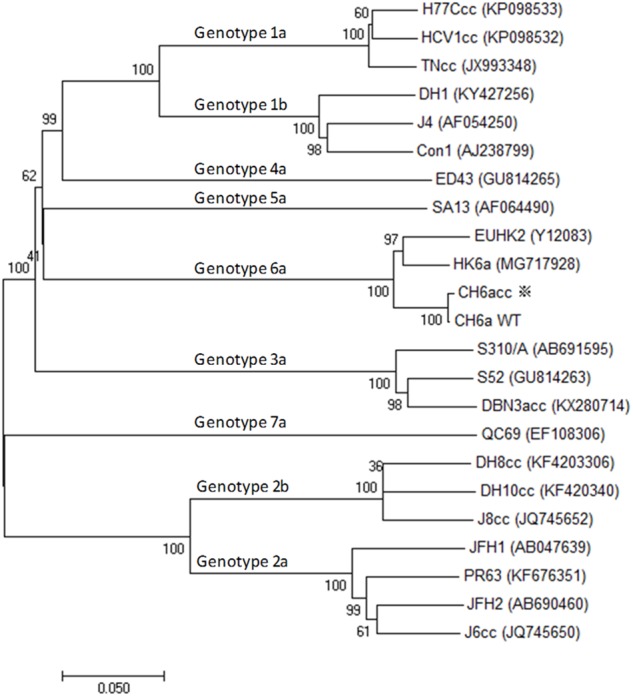
Phylogenetic analysis of CH6a and other HCV isolates of genotypes 1–7. Phylogenetic analysis of ORF nucleotide sequences of CH6a wild-type (WT) and CH6acc (asterisk), as well as other HCV isolates representative of the advanced cell culture systems of respective genotype, including cell culture infectious full-length clones and those full-length sequences that are not infectious *in vitro*. The available culture infectious full-length HCV clones are 1a (TNcc, HCV1cc, and H77Ccc) (Li et al., 2012b; Li et al., 2015b), 2a (JFH1, J6cc, PR63, and JFH2) (Wakita et al., 2005; Date et al., 2012; Li et al., 2012a; Lu et al., 2014), 2b (J8cc, DH8cc, and DH10cc) (Ramirez et al., 2014), 3a (DBN3acc) (Ramirez et al., 2016), and 6a [recently published HK6acc (Pham et al., 2018), as well as CH6acc reported in this study]. Full-length sequence includes 1b (Con1, J4, and DH1), 3a (S52), 4a (ED43) and 5a (SA13), and 7a (QC69) (Gottwein et al., 2009; Li Y.P. et al., 2014; Pham et al., 2017; Li et al., 2018). The evolutionary history was inferred by using the neighbor-joining method in the freeware Molecular Evolutionary Genetics Analysis (MEGA), version 7 (Kumar et al., 2016).

To develop a novel culture infectious clone for HCV genotype 6a isolate, we selected a genotype 6a-infected serum with high viral RNA load of 1.2×10^7^ international units (IU)/ml. The HCV genome was determined using five overlapping RT-PCRs covering nucleotides 60-9570 [nucleotides 60-3589, 3065-5892, 5510-7942, 7604-9403, and 8982-9570; primers are in Supplementary Table S1; corresponding to nts 62-9575 of strain H77 (GenBank number AF009606)]. The PCR fragments were TA-cloned, and 6–12 clones of each PCR product were sequenced to obtain a consensus sequence. One clone of each PCR region that shared the highest homology to the consensus sequence was selected to assemble a full-length cDNA clone of 9641 nucleotides, in which the nucleotides 1–59 and 9571–9641 were taken from strain HK6a (KF134011) (Li et al., 2011a; Li Y.P. et al., 2014) and the conserved 3′X-tail of strain H77 (AF009606) (Kolykhalov et al., 1996, 1997), respectively. The NS5B sequence was further confirmed and optimized by additional 12 clones made from independent RT-PCRs and cloning analyses. The sequence spanning the variable region, polyU/UC tract, and partial 3′X-tail of the 3′UTR, was determined by clonal analysis of 5 clones. The final full-length ORF was highly homologous to the consensus sequence, but differed by 54 nucleotides and two amino acids S2362G (“S” in consensus to “G” in the assembled clone; corresponding to nucleotide change G7426A) and N2738D (A8554G). The full-length CH6a cDNA was constructed into pGEM-9Zf(-) vector immediately downstream of T7 promoter and designed pCH6aFL. The ORF of CH6a encodes a polyprotein of 3019 amino acids and differs from isolate EUHK2 (Y12083) by 6% at nucleotide and amino acid sequences (Adams et al., 1997). The CH6a has one extra amino acid at aa476 compared to EUHK2 polyprotein. For Core-NS5A region, CH6a differs from HK6a (KF589889) by 6 and 3% at the nucleotide and amino acid levels, respectively (Figure 1), and HK6a has one extra amino acid at aa235 position.

### Adaptation of CH6a Core-NS2 Recombinant, Containing JFH1 5′UTR and NS3-3′UTR Sequences, Identified New Adaptive Mutations

We aimed to develop an infectious cell culture system for full-length HCV genotype 6a clone. We initially tested the viability of full-length CH6a clone (CH6aFL) in Huh7.5 and Huh7.5.1 cells by transfection of *in vitro*-transcribed RNA transcripts. In three independent transfections in each cell line, we did not detect any HCV-positive cells in immunostaining for HCV Core and NS5A proteins after 21–28 days of follow up. Thus, the CH6aFL was non-viable *in vitro*.

Previously, we identified LSG mutations F1469L/A1677S/ D2987G (corresponding to F1464L/A1672S/D2979G by H77 aa positions) that enabled the replication of full-length HCV genotypes 1a, 2a, and 2b (Li et al., 2012a,b; Ramirez et al., 2014; Li C. et al., 2014; Li Y.P. et al., 2014). LSG plus additional five mutations could adapt 5′UTR-NS5A (5-5A) recombinant of the other 6a isolate, HK6a, with JFH1 NS5B-3′UTR (Li Y.P. et al., 2014). Here, we introduced LSG into the CH6aFL to make CH6aFL_LSG. We also constructed CH6a 5-5A and Core-NS5A (C-5A) recombinants containing LS mutations; G mutation was not included as it locates in JFH1 NS5B region of the recombinants. In three transfections, all of three recombinants showed no evidence of HCV replication by anti-HCV Core antibody immunostaining throughout 28 days of follow up (Supplementary Table S2). Next, we combined LS with additional mutations V1555L/I1720F/L1795M from HK6a 5-5A virus (Li Y.P. et al., 2014) and K1303R/K1696R from another consensus 6a subgenomic replicon (Yu et al., 2014) to make 5-5A_7m and C-5A_7m (Supplementary Table S2). In three transfections of Huh7.5.1 cells, both 5-5A_7m and C-5A_7m showed low level of viral replication, being 1 and 10% HCV-positive cells, respectively. However, the viruses did not spread after 28–35 days of follow up. We made more efforts to test additional eight C-5A recombinants, which contained different combinations of mutations selected from HK6a 5-5A viruses (Li Y.P. et al., 2014) and/or from the 6a subgenomic replicon (Yu et al., 2014), however, none had a productive HCV replication (Supplementary Table S2). Additionally, we also tested CH6a C-NS3 or CH6a C-NS2 plus NS4A-NS5A recombinants containing selected mutations (other regions of these recombinants were JFH1), but both showed only 1–10% HCV-positive cells and did not spread after 35–41 days (Supplementary Table S2).

**FIGURE 2:**
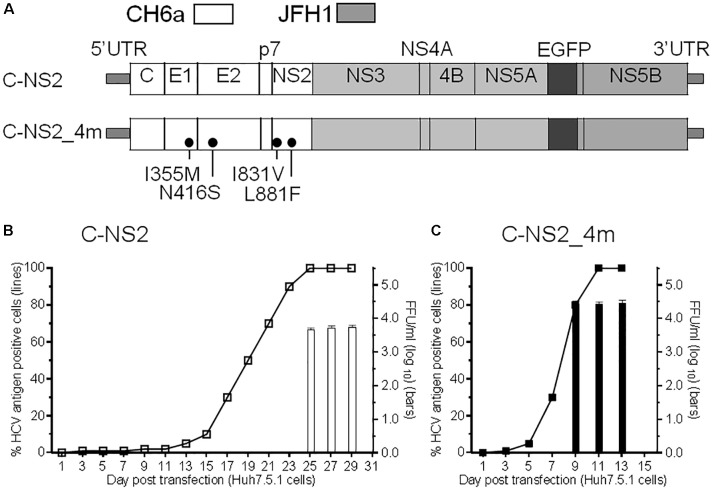
Culture adaptation of CH6a Core-NS2 recombinant identified mutations that enhanced virus production. RNA transcripts of CH6a C-NS2 recombinants (wild-type or with mutations as indicated in panel **A**) were transfected into Huh7.5.1 cells, either HCV Core or NS5A-EGFP antigens were detected by immunostaining or directly visualizing under fluorescence microscope, respectively. Percentage of HCV-positive cells was estimated (left *y*-axis; shown as line plots). HCV infectivity titers in the supernatant at peak of infection (≥80% HCV-positive cells) were determined by focus-forming-unit (FFU) assay [mean of triplicate infections ± standard deviation (SD), right y-axis; shown as bar graphs]. **(B)** Transfection of wild-type CH6a C-NS2 recombinant. The supernatant of C-NS2 virus collected from days 25 and 27 were inoculated to naïve cells to generate passage virus, and consecutively we generated the fifth- and ninth-passage viruses for sequence analysis (Table 1). **(C)** Transfection of CH6a C-NS2_4m, which contained four mutations (4m, I355M/N416S/I831V/L881F) identified in the C-NS2 virus of the fifth-passage (Table 1).

Since we failed to recover virus from the full-length and recombinants with CH6a C-5A, C-NS3, or C-NS2 plus NS4A-NS5A, we stepped back to construct a CH6a C-NS2 recombinant in the backbone of J6/JFH1-NS5AΔ40-EGFP, a 2a chimera expressing EGFP in NS5A for the convenience of monitoring HCV infection (Gottwein et al., 2011a) (Figure 2A). J6/JFH1-NS5AΔ40-EGFP was comparable to J6/JFH1 in transfection cultures (Gottwein et al., 2011a). In RNA transfection of Huh7.5.1 cells, C-NS2 recombinant showed 1% HCV-positive cells at day 1 and spread to peak infection (≥80% of cultured cells) at day 23 post transfection. To determine the peak infectivity titers, which largely correlated with ≥80% HCV-positive cells in the culture, we titrated the supernatant collected from 2–3 time points at peak infection (also applied to other recombinants throughout this study), as previously described (Li et al., 2012a,b; Li Y.P. et al., 2014; Li et al., 2015b). It should be noted that lower infectivity titers may have been produced at intermediate time points along with increasing percentage of infected cells (Li Y.P. et al., 2014). The C-NS2 produced peak infectivity titer of 10^3.8^ FFU/ml (Figure 2B). The supernatant from peak infections was inoculated to naïve cells and allowed the virus spreading to peak infection to obtain the first-passage virus. Consecutively, we generated the ninth-passage virus, and we sequenced the fifth- and the ninth-passage viruses and identified a number of mutations (Table 1). Addition of I355M/N416S/I831V/L881F (4m, completed changes in the fifth-passage virus) into C-NS2 recombinant accelerated virus spread, as C-NS2_4m reached peak infection at day 9 post transfection and released supernatant infectivity titers up to 10^4.6^ FFU/ml (Figure 2C). Thus, the 4m mutations have an adaptation effect for CH6a Core-NS2 recombinant.

**Table 1:** Sequence analysis of CH6a Core-NS2 recombinants identified additional mutations.

HCV	Passage (day)	E1	E2	E2	E2	E2	p7	NS2	NS2	NS2	NS2
**Nucleotide position**											
Recombinant specific		1405	1587	1657	2220	2578	2680	2831	2981	3145	3337
H77 reference (AF009606)		1406	1591	1661	2206	2564	2666	2815	2965	3131	3323
Recombinant nucleotide		A	A	A	T	A	T	A	C	G	T
CH6a Core-NS2 recombinant											
	Fifth (14)	G	G	⋅	⋅	⋅	G	G	T	⋅	⋅
	Ninth (10)	G	G	G	C/T	G	G	G	T	T	C
**Amino acid position**											
Recombinant specific		355	416	439	627	746	780	831	881	935	999
H77 reference (AF009606)		355	417	440	622	741	775	825	876	930	994
Amino acid change		I-M	N-S	S	V-A	I-M	A	I-V	L-F	G	G

*Culture supernatant from CH6a Core-NS2 (C-NS2) transfected Huh7.5.1 cells (days 25 and 27, Figure 2B) was inoculated to naïve cells and the supernatant was collected when the virus spread to most of cultured cells to obtain the first-passage virus. We consecutively passaged the virus and generated the ninth-passage virus. Sequence analysis of Core-NS2 region of the fifth- and ninth-passage viruses identified additional mutations. Nucleotide and amino acid positions of the C-NS2 recombinant and the corresponding position of H77 reference sequence (AF009606) are given. Two capital letters separated by a slash indicates a nucleotide quasispecies (50/50) in sequencing reads. Dots (⋅) indicate identity with original nucleotide, hence the original amino acid was accordingly shown in a single letter. Four mutations (4m, I355M/N416S/I831V/L881F) were engineered into the C-NS2 to make C-NS2_4m (Figure 2)*.

### Efficient CH6a Core-NS5A Recombinants Required Combinations of Mutations

**FIGURE 3:**
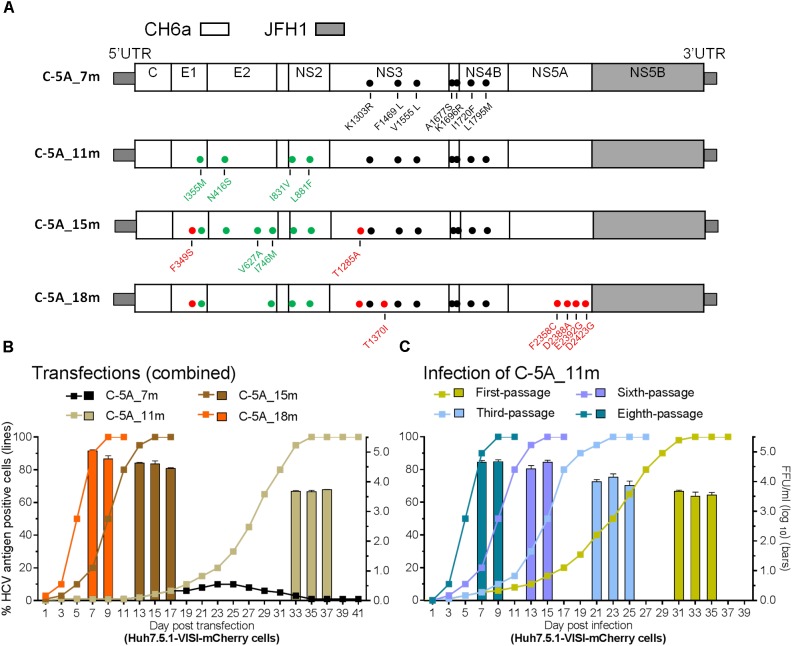
Combinations of mutations promoted CH6a Core-NS5A recombinants to replicate efficiently in cultured cells. **(A)** Schematic diagrams of CH6a C-5A recombinants with different mutations (7m, 11m, 15m, and 18m). 7m was combinations of F1469L/A1677S (LS) (Li et al., 2012a), V1555L/I1720F/L1795M from HK6a 5-5A virus (Li Y.P. et al., 2014), and K1303R/K1696R from the consensus 6a subgenomic replicon (Yu et al., 2014). 4m (I355M/N416S/I831V/L881F; in green color) was identified from the Core-NS2 recombinant (Table 1). Mutations in red color were identified from the passaged C-5A_11m viruses (Table 2). **(B)** RNA transcripts of C-5A recombinants with indicated mutations were transfected into Huh7.5.1-VISI-mCherry cells (Zhao et al., 2017), and the percentage of mCherry-positive cells was estimated (left *y*-axis; lines). HCV infectivity titers in culture supernatants at peak infection were determined (mean of triplicate infections ± SD, right *y*-axis; bars). Transfections were done in different experiments, and the data were combined in the graph. **(C)** C-5A_11m virus collected from days 33, 35, and 37 (in panel **B**) were serially passaged to naïve cells. Virus spread and peak infectivity titers for passages 1, 3, 5, and 8 are shown for one of two independent infections performed in parallel; similar virus spread and infectivity titers were recorded for the other infected culture. ORF sequence analyses of the passaged viruses collected from peak infections are shown in Table 2.

Since the 4m apparently enhanced the viability of C-NS2 virus (Figure 2) and C-5A_7m showed more HCV-positive cells than 5-5A_7m (above), we tested the effect of 4m in C-5A_7m by making C-5A_11m recombinant (Figure 3A). For the convenience of monitoring HCV infection, we used Huh7.5.1-VISI-mCherry cells for transfection and infection in the following experiments (Zhao et al., 2017), unless otherwise stated. Similar to the results from Huh7.5.1 cells, C-5A_7m recombinant showed only 1–10% HCV-positive Huh7.5.1-VISI-mCherry cells and did not spread after 41 days (Figure 3B). Addition of 4m to C-5A_7m accelerated the virus spread, as C-5A_11m spread to peak infection at day 31 and released peak infectivity titers of 10^3.8^ FFU/ml (Figure 3B). We continuously passaged C-5A_11m virus up to eight passages in two independent cultures, and the virus spread and the infectivity titers in two cultures were similar and improved along with the increasing rounds of passages. The virus spread was accelerated and the time of reaching peak infection was reduced from 31 days in the first-passage to 7 days in the eighth-passage, and concomitantly the peak infectivity titers were increased from 10^3.7^ to 10^4.8^ FFU/ml (Figure 3C). ORF sequence analysis of the first-, third-, sixth-, and eighth-passage-recovered viruses identified a number of mutations in Core-NS5A regions (Table 2). We added more mutations that identified in C-NS2 and C-5A_11m viruses by different combinations into C-5A_11m to construct C-5A_15m and C-5A_18m (Figure 3A and Supplementary Table S2). The C-5A_15m contained the 11m plus F349S/T1285A (found in the passaged C-5A_11m from two independent infection experiments, Table 2) and V627A/I746M (found in the ninth-passage C-NS2 virus, Table 1), while the C-5A_18m contained 11m plus seven mutations (F349S/T1285A/T1370I/F2358C/D2388A/E2392G/D2423G) from the passaged C-5A_11m viruses (Table 2). In two RNA transfections, both C-5A_15m and C-5A_18m spread efficiently in transfection cultures and reached peak infection at days 13 and 7, with peak infectivity titers of 10^4.8^ and 10^5.1^ FFU/ml, respectively (Figure 3B). These results demonstrate that the combination of adaptive mutations identified from various HCV recombinants, such as 18m, could adapt CH6a Core-NS5A recombinant to replicate efficiently in cultured cells.

**Table 2:** Sequence analysis of CH6a C-5A_11m virus that went through a number of passages.

HCV	Passage (day)	Core	Core	E1	E1	NS2	NS2	NS3	NS3	NS3	NS4B	NS5A	NS5A	NS5A	NS5A	NS5A	NS5A	NS5B
***Part I, CH6a C-5A_11m^a^, independent infection experiment 1***								
**Nucleotide position**																		
Recombinant specific		346	391	1225	1386	2838	3134	4193	4423	4449	5983	6338	6787	7000	7213	7413	7515	8962
H77 reference (AF009606)		347	392	1226	1387	2824	3120	4179	4409	4435	5969	6324	6773	6986	7199	7394	7483	8939
Recombinant nucleotide		C	T	T	T	T	A	A	C	C	C	T	A	T	C	T	T	A
CH6a C-5A_11m^a^, exp. 1																		
	First (35)	⋅	⋅	⋅	⋅	⋅	⋅	G	A	⋅	⋅	⋅	⋅	⋅	⋅	⋅	⋅	⋅
	Third (25)	⋅	⋅	⋅	C	⋅	⋅	G	A	⋅	⋅	⋅	⋅	⋅	⋅	⋅	⋅	⋅
	Sixth (14)	⋅	C		C	C	G	G	A	T	T	⋅	⋅	C	T	G	G	⋅
	Eighth (7)	T/t	C	C	C	C	G	G	A	T	T	C	G	C	T	G	G	G
**Amino acid position**																		
Recombinant specific		2	17	295	349	833	932	1285	1361	1370	1881	2000	2149	2220	2291	2358	2392	2874
H77 reference (AF009606)		2	17	295	349	828	927	1280	1356	1365	1876	1995	2144	2215	2286	2351	2381	2866
Amino acid change		S	R	P	F-S	I-T	R-G	T-A	T	T-I	T	L	E	C	L	F-C	E-G	M-V

**HCV**	**Passage (day)**	**E1**	**E1**	**E1**	**E2**	**E2**	**NS2**	**NS3**	**NS3**	**NS3**	**NS3**	**NS3**	**NS3**	**NS4A**	**NS4B**	**NS5A**	**NS5A**

***Part II, CH6a C-5A_11m^a^, independent infection experiment 2***								
**Nucleotide position**																	
Recombinant specific		1000	1078	1386	1708	2162	2791	3976	4115	4193	4423	4899	5260	5394	5905	7503	7608
H77 reference (AF009606)		1001	1079	1387	1712	2148	2777	3962	4101	4179	4409	4885	5246	5380	5891	7469	7585
Recombinant nucleotide		C	A	T	C	T	T	T	T	A	C	T	A	T	T	A	A
CH6a C-5A_11m^a^, exp. 2																	
	First (35)	⋅	⋅	⋅	⋅	⋅	⋅	⋅	⋅	G	A	⋅	⋅	⋅	⋅	⋅	⋅
	Third (25)	⋅	⋅	⋅	⋅	C	⋅	⋅	⋅	G	A	⋅	⋅	⋅	⋅	⋅	⋅
	Sixth (14)	T	G	⋅	A	C	G	C/T	C	G	A	C	G	C	C/T	C	G
	Eighth (7)	T	G	C/T	A	C	G	C	C	G	A	C	G	C	C	C	G
**Amino acid position**																	
Recombinant specific		220	246	349	456	608	817	1212	1259	1285	1361	1520	1640	1685	1855	2388	2423
H77 reference (AF009606)		220	246	349	457	603	812	1207	1254	1280	1356	1515	1635	1680	1850	2376	2415
Amino acid change		I	L	F-S	A	L	N-K	S	L	T-A	T	V-A	V	V-A	D	D-A	D-G

*Culture supernatant from CH6a Core-NS5A_11m (C-5A_11m) transfected Huh7.5.1-VISI-mCherry cells (days 33, 35, and 37; Figure 3B) was passaged to naïve cells in two independent infection experiments (experiments 1 and 2), and consecutively we generated the eighth-passage virus. ORF sequence analysis of the first-, third-, sixth-, and eighth-passage viruses revealed a number of mutations in two passaged viruses (Part I for infection experiment 1; Part II for infection experiment 2). One capital letter and a lowercase letter separated by a slash indicate a nucleotide quasispecies of dominant/minor in sequencing reads. See the Table 1 Legend for nucleotide annotations. ^a^11m refers to the combination of mutations I355M/N416S/I831V/L881F/K1303R/F1469L/V1555L/A1677S/K1696R/I1720F/L1795M*.

### Robust CH6a Full-Length Infectious Clones With Combined Mutations

During the course of adaptation of C-5A_11m virus by serial passages, more mutations were accumulated (Table 2). In parallel, we also tested the effect of 11m mutations in CH6a recombinant with both 5′UTR and 3′UTR from JFH1 (designated CH6aORF_11m) or with only 5′UTR from JFH1 (CH6aCore-3′UTR), however, both recombinants displayed a very low level of infection without virus spread. Five CH6aORF or CH6aFL recombinants with NS5B region from different clones were also tested, but none showed a higher infection (Supplementary Figure S1). Next, we attempted to adapt CH6aORF_11m by selectively adding the C-5A_11m-recovered mutations and other mutations. Of which, addition of T1285A (from the first-passage C-5A_11m, Table 2) plus D2987G/Y2989F (“GF” mutations, important for 1a, 2a, and 2b clones) (Li et al., 2012a,b; Ramirez et al., 2014; Li Y.P. et al., 2014) or T1285A/D2987G/Y2989F plus F349S (from the third-passage C-5A_11m, Table 2) replicated at a low level but did not achieve virus spread in three transfections. The CH6aORF or CH6aFL recombinants with different mutations tested were listed in Supplementary Figure S1.

**FIGURE 4:**
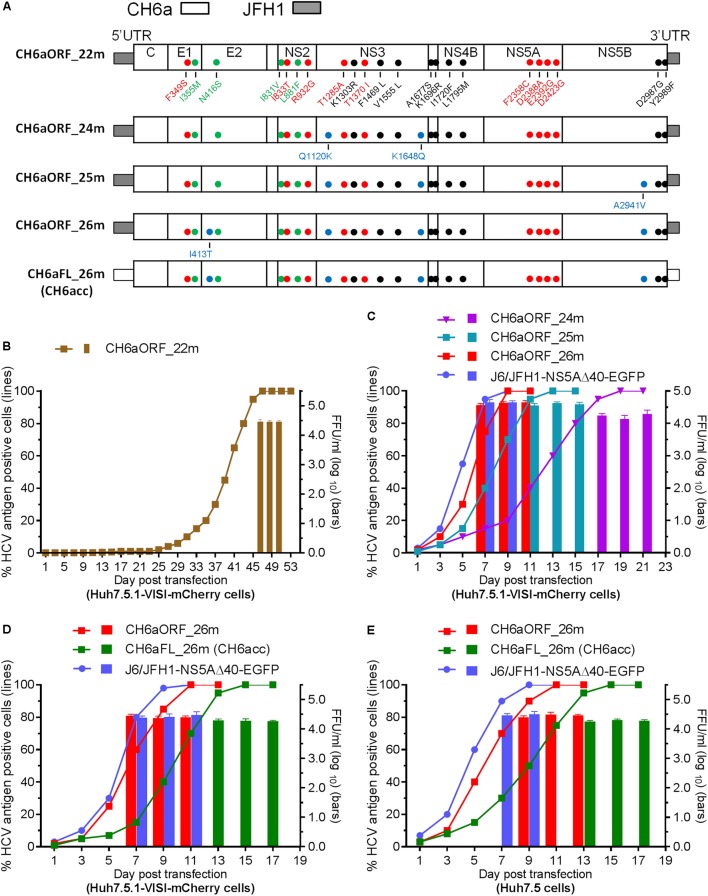
CH6a ORF and full-length infectious clones replicated efficiently in transfection cultures. **(A)** Schematic diagrams of CH6a ORF and full-length recombinants (CH6aORF and CH6aFL) with 22, 24, 25, and 26 mutations (22m, 24m, 25m, and 26m). The 22m was the combination of 18m (Figure 3A) plus I833T/R932G (from C-5A_11m viruses) (Table 2) and D2987G/Y2989F (GF mutations) (Li et al., 2012a). Mutations Q1120K/K1648Q, Q1120K/K1648Q/A2941V, and I413T/Q1120K/K1648Q/A2941V identified from CH6aORF_22m (in **B** and Table 3) were engineered into the CH6aORF_22m to make CH6aORF_24m, CH6aORF_25m, and CH6aORF_26m, respectively. Mutations were indicated by colors [black, mutations identified previously; green, 4m from C-NS2 virus (Table 1); red, C-5A_11m virus (Table 2); and blue, CH6aORF_22m (Table 3)]. **(B–D)** Transfection of CH6a ORF and full-length recombinants with different mutations as indicated. The percentage of mCherry-positive cells was estimated (left *y*-axis; lines) and HCV infectivity titers at peak infections were determined (mean of triplicate infections ± SD, right *y*-axis; bars). The efficient J6/JFH1-NS5AΔ40-EGFP was used as positive control (Gottwein et al., 2011a). **(E)** Transfections of CH6aORF_26m and CH6aFL_26m (CH6acc) in Huh7.5 cells. The HCV-positive cells were visualized by anti-HCV Core immunostaining (left *y*-axis; lines), and HCV infectivity titers were done by FFU assay (mean of triplicate infections ± SD, right *y*-axis; bars).

Since CH6a C-5A_18m replicated efficiently in cultures, we introduced 18m plus I833T/R932G (found in the sixth- and eighth-passage C-5A_11m viruses) (Table 2) and D2987G/Y2989F (Li et al., 2012a,b; Ramirez et al., 2014; Li Y.P. et al., 2014), into the CH6aORF to generate CH6aORF_22m (Figure 4A). In addition, we also introduced 15m (the “15m” in C-5A_15m virus, Figure 3) plus D2423G/D2987G/Y2989F or F2358C/D2388A/E2392G/D2423G/D2987G/Y2989F into the CH6aORF (CH6aORF_18m and CH6aORF_21m in Supplementary Figure S1). In transfection cultures, all three recombinants showed 1–5% HCV-positive cells, but CH6aORF_22m spread faster and peaked at day 47, releasing peak HCV titers of 10^4.4^ FFU/ml (Figure 4B). Other combinations of mutations were also tested, but the replication was very low and the viruses did not spread in the cultures (Supplementary Figure S1).

We passaged the transfection recovered CH6aORF_22m. ORF sequence analysis of the first- and the second-passage CH6aORF_22m viruses identified I413T in E2, Q1120K/K1648Q in NS3 and A2941V in NS5B (Table 3). We engineered Q1120K/K1648Q, Q1120K/K1648Q/A2941V, and I413T/Q1120K/K1648Q/A2941V back into the CH6aORF_22m to make CH6aORF_24m, CH6aORF_25m, and CH6aORF_26m, respectively (Figure 4A). In RNA transfected cultures, CH6aORF_26m was the most efficient and spread to peak infection at day 7 with peak infectivity titers of 10^4.6^ FFU/ml, comparable to the positive control J6/JFH1-NS5AΔ40-EGFP [the infectivity was equivalent to J6/JFH1 (Gottwein et al., 2011a)]. The virus spread of CH6aORF_25m and CH6aORF_24m were slightly delayed and reached peak infection at days 9 and 15 with peak infectivity titers of 10^4.2^–10^4.3^ FFU/ml (Figure 4C). Sequence analysis of the second-passage CH6aORF_26m revealed that no additional mutation was required (Table 3), thus CH6aORF_26m was genetically stable. Next, we tested 26m in full-length CH6aFL genome. CH6aFL_26m was slightly attenuated compared to CH6aORF_26m and spread to most of cultured cells at day 13, producing peak HCV titers of 10^4.3^ FFU/ml (Figure 4D). In the second-passage CH6aFL_26m virus, all engineered mutations were maintained and two quasispecies L1700L/M and T1823T/S were identified.

**Table 3:** Sequence analyses of CH6aORF_22m virus after the first and second passages.

HCV	Passage (day)	E2	NS3	NS3	NS5B	NS5B	NS5B
**Nucleotide position**							
Recombinant specific		1582	3698	5282	7997	8099	9162
H77 reference (AF009606)		1578	3684	5268	7974	8076	9138
Recombinant nucleotide		T	C	A	G	C	G
CH6aORF_22m recombinant^a^							
	First (10)	C/t	A	C	⋅	T	T
	Second (7)	C	A	C	A/G	T	T
CH6aORF_26m	Second (5)	C	A	C	⋅	⋅	T
**Amino acid position**							
Recombinant specific		413	1120	1648	2553	2587	2941
H77 reference (AF009606)		414	1115	1643	2545	2579	2933
Amino acid change		I-T	Q-K	K-Q	D-N	L	A-V

*Culture supernatant from CH6aORF_22m (Figure 4A) transfected Huh7.5.1-VISI-mCherry cells was passaged to naïve cells. The first- and second-passage viruses were sequenced for the ORF. Mutations identified in the viruses are listed. Two capital letter separated by a slash indicates a nucleotide quasispecies of 50/50 in sequencing reads. Dot (⋅), indicates no change for the original nucleotide. Nucleotides in shading indicate that these nucleotides were engineered into the recombinant plasmid. Sequence analysis of CH6aORF_26m revealed that no additional mutations were identified. ^a^Four complete coding changes (I413T/Q1120K/K1648Q/A2941V) were engineered into Ch6aORF_22m to make CH6aORF_26m (Figure 4A)*.

Next, we examined the viability of CH6aORF_26m and CH6aFL_26m in Huh7.5 cells (Figure 4E). In RNA transfections, CH6aORF_26m reached peak infection at day 7 and released infectious virus particles of 10^4.5^ FFU/ml, whereas CH6aFL_26m peaked at day 11 and produced supernatant HCV of 10^4.3^ FFU/ml. To confirm the transfection-recovered viruses, we sequenced the ORF of second-passage viruses. The engineered 26m was maintained in both viruses. Thus, we have developed an efficient cell culture system for HCV genotype 6a isolate CH6a. Here we designate the full-length recombinant CH6aFL_26m as “CH6acc” (for “CH6a cell culture-derived”; GenBank no. MH155319).

### Mutations Newly Identified Were Important for the Viability of CH6acc Virus

**FIGURE 5:**
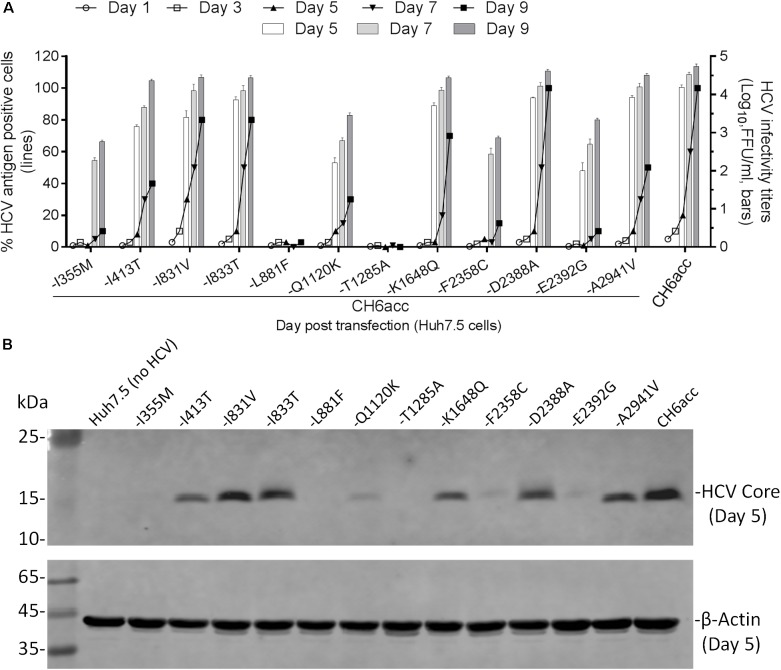
Effects of newly identified adaptive mutations on the viability of CH6acc. **(A)** RNA transcripts of CH6acc and CH6acc with each of 12 putative adaptive mutations, which were newly identified in this study, mutated back to the wild-type sequence were transfected into Huh7.5 cells. Virus spread (% HCV antigen- positive cells) was determined by anti-HCV Core immunostaining (left *y*-axis; lines), and HCV infectivity titers (FFU/ml) in supernatants from cultures at day 5, 7, and 9 were determined and shown as the mean from triplicate infections ± SEM (the standard error of the mean). The transfection experiments were performed in two independent experiments, and the representative data from one experiment were shown. **(B)** Intracellular HCV Core antigen levels were determined by Western blotting. Cell lysates were separated through acrylamide gels, and proteins were transferred to PVDF membranes and immunoblotted with anti-HCV Core C7-50 antibody for detection of HCV Core and anti-β-actin for detection of host cellular actin.

In the process of developing CH6acc, we found that mutations identified previously enabled the replication of C-5A recombinant (C-5A_7m in Figure 3). These mutations were acquired from other replicon or infectious recombinants and have also been demonstrated to stably maintain in the genome and to be important for RNA replication and virus production (Li et al., 2012a; Li Y.P. et al., 2014; Yu et al., 2014; Pham et al., 2018). On the basis of C-5A_7m, other mutations identified from C-NS2, C-NS5A, CH6aORF, or full-length recombinants were added to finally develop CH6acc. Here, we tested the necessity of the mutations identified in this study and have not been investigated elsewhere. We mutated CH6acc at each of the twelve mutations I355M, I413T, I831V, I833T, L881F, Q1120K, T1285A, K1648Q, F2358C, D2388A, E2392G, and A2941V back to the original sequence and named the resultant mutants by “-mutation.” We tested the viability of these mutants in transfections of Huh7.5 cells by monitoring virus spread within 9 days and titrated the supernatant infectivity for days 5, 7, and 9 (Figure 5A). As expected, CH6acc spread efficiently, reached peak infection at day 9, and produced infectivity titers of 10^4.8^ FFU/ml. -L881F and -T1285A viruses were severely attenuated and did not spread after 9 days, releasing no supernatant infectivity titers. -I355M, -Q1120K, -F2358C, and -E2392G were attenuated to some extents, with the delayed virus spread (10–30% at day 9) and the lower infectivity titers (being 1.5–2.0 log_10_FFU/ml lower than CH6acc); the infectivity titers of -I355M and -F2358C at day 5 were undetectable. -I413T was slightly attenuated (40% at day 9), while other mutated viruses were not apparently affected. We also determined the intracellular HCV Core levels at day 5 post transfection for these mutants and found that the results resembled the observations for the virus spread and the infectivity titers (Figure 5B). Together, these results indicate that the absence of each of mutations I355M, L881F, Q1120K, T1285A, F2358C, and E2392G apparently affected the viability of CH6acc, of which L881F and T1285A had the greatest impact, thus showing the importance of these mutations in the viability of CH6acc.

### Novel Adaptive Mutations Important for Viral Replication, Assembly, and Release

**FIGURE 6:**
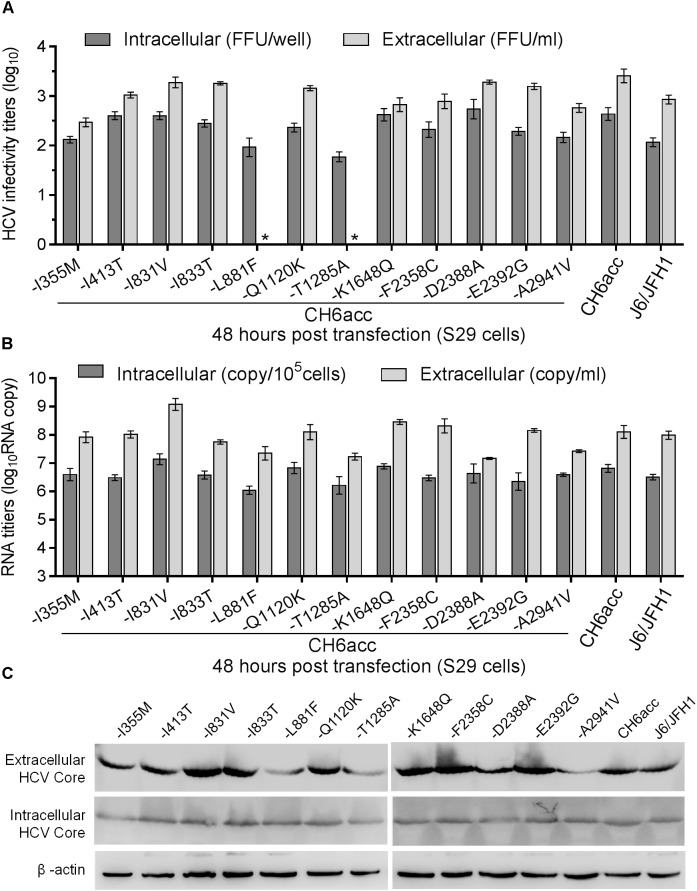
Functional analysis of the role of newly identified CH6acc adaptive mutations in the HCV life cycle. Equal amount of RNA transcripts (5 μg) from CH6acc and twelve CH6acc mutants with single mutation changed back to original sequence were transfected into HCV entry-deficient S29 cells (Russell et al., 2008). Cell lysates (intracellular) and culture supernatant (extracellular) were collected 48 h post transfection. HCV infectivity titers, RNA copies, and Core levels were determined and normalized to replication-independent genome, J6/JFH1-GND. **(A)** Intracellular and extracellular HCV infectivity titers. ^∗^, no FFU was detected by manual count. Values are expressed as log_10_FFU/ml (45 μl loaded) for extracellular titers and as log_10_FFU/well (1/8 cell lysate of a well of 6-well-plate) for intracellular infectivity titers. **(B)** HCV RNA levels in the cells and supernatant. The method for the determination of RNA levels were as described previously (Boson et al., 2011). **(C)** HCV Core levels. Western blotting was performed on the cell lysates and supernatant harvested 48 h post transfection.

To address the role of those 12 newly identified CH6acc adaptive mutations in the viral life cycle, we performed a single-cycle production assay using Huh7-derived S29 cells, a cell line deficient for the HCV entry receptor CD81 (Russell et al., 2008). We tested the mutants -I355M, -I413T, -I831V, -I833T, -L881F, -Q1120K, -T1285A, -K1648Q, -F2358C, -D2388A, -E2392G, and -A2941V in transfections of S29 cells. After transfection, the intracellular and extracellular infectivity titers were determined at 48 h (Figures 6A,B). The HCV Core levels were visualized by Western blotting (Figure 6C). -L881F and -T1285A mutants showed a lower intracellular titer of approximately 1 log_10_FFU/well than CH6acc virus and were undetectable for extracellular titers (Figure 6A). Both intracellular and intracellular RNA titers of these two recombinants were also lower by around 1 log_10_(RNA copy) than that of CH6acc virus (Figure 6B). Other mutants showed little difference from CH6acc virus in the single-cycle short-term assay. Thus, each of L881F and T1285A was essential for the virus replication and release. Together with the results from Huh7.5 transfections (Figure 5), we conclude that L881F and T1285A were required for HCV RNA replication and virus release, whereas I355M, I413T, Q1120K, F2358C, E2392G, and A2941V might involve in other steps of viral life cycle, most likely affecting virus assembly, release, and/or spread.

### Advantage of HCV Clone Assembled Using PCR Fragments Shared the Highest Homology to the Consensus Sequence

**FIGURE 7:**
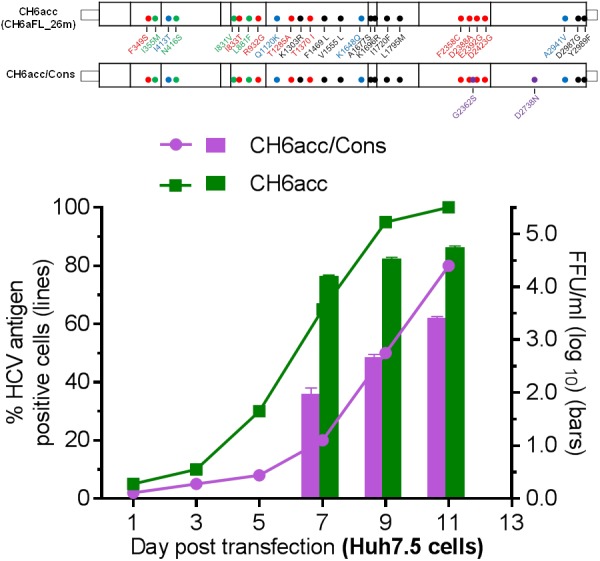
CH6a full-length clone assembled from the consensus-like PCR fragments was more efficient in virus production. Two amino acids, S2362G in NS5A and N2738D in NS5B of CH6acc genome were mutated to the consensus sequence, designated CH6acc/Cons. RNA transcripts were transfected into Huh7.5 cells, and CH6acc was included in parallel. CH6acc/Cons was delayed in virus spread and attenuated in infectivity titers. HCV-positive cells were visualized by secure anti-HCV Core immunostaining (left *y*-axis; lines), and HCV infectivity titers were done by FFU assay (mean of triplicate infections ± SEM, right *y*-axis; bars). In other transfections, CH6aFL/Cons recombinant was non-viable.

The CH6aFL genome was assembled by using PCR product-derived clones that shared the highest homology to the consensus sequence and the final CH6aFL cDNA clone differed from the consensus sequence by two amino acids, S2362G in NS5A and N2738D in NS5B. Both regions are known to be important for replication and infectious virus production of HCV. Thus, we examined whether these two amino acids were important for the viability of the virus. We mutated these two amino acids back to the consensus sequence to make CH6aFL/Cons and CH6acc/Cons. In transfected cultures, CH6aFL/Cons was non-viable, and CH6acc/Cons was attenuated compared to CH6acc (Figure 7). CH6acc/Cons showed delayed virus spread and lower infectivity titers by 30–100-fold at days 7, 9, and 11 post transfection. Thus, G2362 and D2738 in the assembled CH6aFL genome were beneficial for infectious virus production. These results also indicate that assembly of PCR fragments sharing the highest homology to the consensus sequence represents a strategy advantageous for making an infectious HCV clone. This strategy increases the chance that the assembled cDNA clone was an actual genome existing in the infecting quasispecies.

### Infectious CH6a Virus Was Inhibited by DAAs Targeting NS3/4A, NS5A, and NS5B in a Dose-Dependent Manner

**FIGURE 8:**
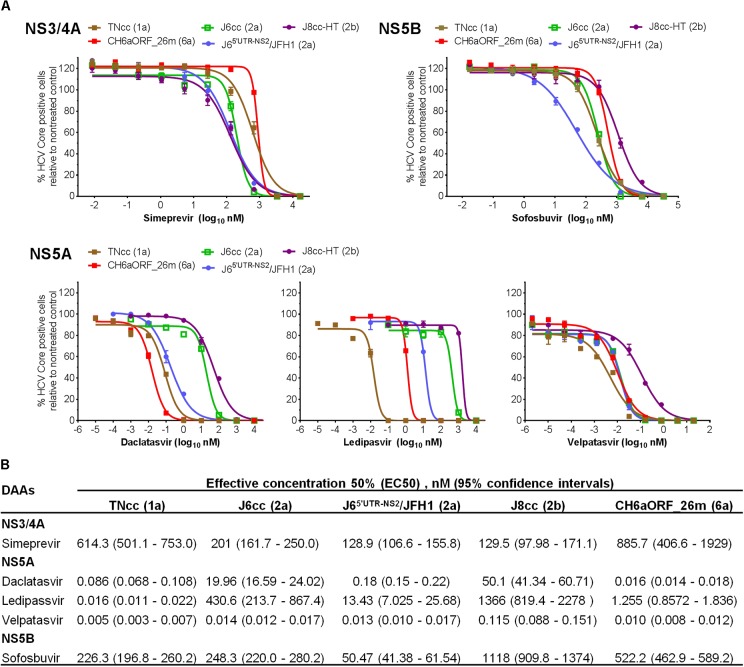
CH6a virus and other full-length infectious HCVcc were inhibited by DAAs targeting NS3/4A, NS5A, and NS5B in a dose- and genotype-dependent manner. **(A)** For treatment experiments of simeprevir and sofosbuvir, Huh7.5 cells in 96-well plates were infected with CH6aORF_26m, in comparison with full-length infectious HCV clones 1a (TNcc), 2a (J6cc), and 2b (J8cc-HT). DAAs were added 24 h post infection and incubated for 48 h. HCV-positive cells were determined 72 h post infection. Genotype 2a chimera J6^5′UTR-NS2^/JFH1 (Li et al., 2011a) was included in the treatments as control. Values are means of triplicates in the experiment ± SEM. For the treatment of NS5A DAAs, Huh7.5 cells with HCV infection at ≥80% were incubated with daclatasvir, ledipasvir, or velpatasvir, and the treatment was left for 48 h. **(B)** EC50 of the DAAs to each virus was calculated from the treatment results shown in **(A)**. Values are means of triplicate determinations, and 95% confidence intervals (CI) are also shown.

As an important application of HCV infectious culture system, we demonstrated the sensitivity of CH6aORF_26m virus to DAAs targeting NS3/4A (simeprevir), NS5A (daclatasvir, ledipasvir, and velpatasvir) and NS5B (sofosbuvir) (Figure 8A). For comparison, we also included full-length infectious clones of genotype 1a (clone TNcc), 2a (J6cc), and 2b (J8cc-HT) (Li et al., 2012a,b; Ramirez et al., 2014). A 2a chimera J6^5′UTR-NS2^/JFH1 (DAA targets being JFH1 sequences) was also included (Li et al., 2011a). The sensitivity to the DAAs was evaluated by comparison of effective concentration 50% (EC50) value (Figure 8B). To simeprevir, CH6aORF_26m virus was most resistant, showing ∼1, 4, 6, and 6-fold level of resistance than TNcc, J6cc, J6^5′UTR-NS2^/JFH1, and J8cc-HT viruses, respectively. For three NS5A inhibitors, the variations of viral sensitivity to daclatasvir and ledipasvir between HCV genotypes were relatively greater than that to velpatasvir. To daclatasvir, CH6aORF_26m was the most sensitive virus, being ∼5, 1200, 11, and 3100-fold more sensitive than TNcc, J6cc, J6^5′UTR-NS2^/JFH1, and J8cc-HT, respectively. To ledipasvir, CH6aORF_26m was 78-fold more resistant than TNcc, but was 340, 10, and 1088-fold more sensitive than genotype 2 viruses J6cc, J6^5′UTR-NS2^/JFH1, and J8cc-HT, respectively. To velpatasvir, the sensitivity of CH6aORF_26m was similar to TNcc, J6cc, and J6^5′UTR-NS2^/JFH1, but was 11-fold more sensitive than J8cc-HT virus. When sofosbuvir was used, CH6aORF_26m virus was more sensitive than J8cc-HT by ∼2-fold, whereas it was more resistant than TNcc, J6cc, and J6^5′UTR-NS2^/JFH1 by ∼2-, 2-, and 10-fold, respectively. These results demonstrate that the efficient infectious culture system of CH6a strain provides a valuable tool for testing of HCV antivirals.

## Discussion

It is still a major challenge to culture HCV *in vitro*, which has become a bottle-neck for HCV research. In this study, we have developed a highly efficient full-length cell culture system for HCV clinical isolate CH6a. We assembled a full-length CH6a genome by using the clones sharing the highest homology with the consensus sequence and adapted this consensus-like clone using mutations identified in the previous and present studies. CH6aORF and CH6aFL full-length recombinants with 26 mutations replicated efficiently following RNA transfection of human hepatoma Huh7.5 cells and Huh7.5.1 cells expressing VISI-mCherry reporter, and both released peak HCV infectivity titers of ∼10^4.6^ FFU/ml. Given the clinical significance of genotype 6 in HCV research, especially its high prevalence in Asian countries, the CH6a infectious clone will be of particular value and provides a useful tool for the studies of this important HCV genotype.

HCV exists as quasispecies in infected patients, which validates the approach that we could develop an infectious clone by introducing certain number of adaptive mutations. Meanwhile, the quasispecies also makes it extremely difficult or impossible to assemble a sequence that represents the dominant or some specific virus population due to the difficulty to amplify the entire genome in a single long PCR amplification. Therefore, a consensus sequence may be artificial and not existing in the pools of infecting genomes. Previously, HCV infectious clones were made based on the synthetic consensus sequences or culture-adapted functional fragments, to which adaptive mutations were subsequently engineered (Li et al., 2012a,b; Ramirez et al., 2014; Li Y.P. et al., 2014; Lu et al., 2014). In this study, we constructed CH6a full-length genome CH6aFL using a different strategy. Initially, we obtained a consensus sequence by alignments of TA-clones of PCR products. The clones shared the highest homology with the consensus were subsequently selected to construct CH6a full-length genome. This strategy minimizes the chance of introducing the nucleotides that are actually in different RNA molecules into the final assembled full-length genome. The CH6a ORF and partial UTRs was assembled using four fragments, and each overlapped with the other upstream and downstream by ∼300–500 nucleotides. The overlapping regions were purposely designed, as it could help to judge that the clones selected for assembly are most likely from one RNA molecule. However, it should be more recommended to amplify the entire or near full-length genome by single long RT-PCR or by less fragments to further minimize the misassembly of the fragments from different RNA molecules. Actually, we succeed in amplifying the nearly entire ORF by one long PCR (data not shown), but the cloning of the long PCR products did not succeed. The final assembled CH6aFL cDNA clone differed from the consensus sequence by two amino acids S2362G (from S in consensus to G in the cloned cDNA; NS5A) and N2738D (NS5B). Through reverse genetics studies, we confirmed that CH6acc was attenuated when these two amino acids were mutated to the consensus sequence (Figure 7). These results suggest that it is an advantage to construct an HCV cDNA clone using PCR-derived fragments sharing high homology with the consensus sequence. Therefore, the success of developing an infectious CH6a genome using the subclones highly homologous to the consensus sequence provides a new strategy for future assembly of full-length HCV genome.

CH6acc contains 26 mutations, of which 9 mutations are previously identified and 17 mutations were acquired in this study. The mutations identified previously are LSGF (F1469L/A1677S/D2987G/Y2989F) from genotype 2a clone J6cc (corresponding to H77 aa1464, 1672, 2979) (Li et al., 2012a), V1555L/I1720F/L1795M from HK6a 5-5A virus (aa1550, 1715, and 1790 in H77) (Li Y.P. et al., 2014), and K1303R/K1696R from the consensus 6a subgenomic replicon (aa1298 and 1691 in H77) (Yu et al., 2014). New mutations found in this study are 4m (I355M/N416S/I831V/L881F) from CH6a C-NS2 virus (Figure 2 and Table 1), C-5A virus F349S/I833T/R932G/T1285A/T1370I/F2358C/D2388A/E2392G/D2423G (Figure 3 and Table 2), and I413T/Q1120K/K1648Q/ A2941V from CH6aORF_22m virus (Figure 4 and Table 3). Besides the cross-genotype effect of LSGF and other previously identified mutations that we selected to use during the adaptation of JFH1-based CH6a recombines, some mutations emerged in this study were also found in other infectious clones. For examples, F349S/T1370I were also identified to be important for two infectious clones of genotype 6a reported recently, HK2cc and HK6acc (Pham et al., 2018). It is notable that F349S (aa349 in H77) was found in three full-length 6a clones (CH6acc, HK2cc, and HK6acc; aa349 corresponding to aa350 in HK6a) [Table 2 and reference (Pham et al., 2018)] and in HK6a C-NS2 virus (Gottwein et al., 2009). F349V was found in DH8cc (Ramirez et al., 2014). In the immediate upstream position, I349M (aa348 in H77) was identified in HK6a 5-5A virus (Li Y.P. et al., 2014). Changes at aa416 (aa417 in H77) was found to be N416T in HK2cc, HK6acc, and HK6a C-NS2 viruses, and importantly the co-presence of F349S and N416T increased the infectivity (Gottwein et al., 2009; Pham et al., 2018). R932G (aa927 in H77) was also found in HK6a 5-5A virus (Li Y.P. et al., 2014). D2423G (aa2415 in H77) was close to the D2424G (aa2416 in H77) in HK2cc and HK6acc (Pham et al., 2018), and E2423G and V2427A in S52 5-5A virus (aa2413 and aa2417 in H77) (Li Y.P. et al., 2014). The mutations identified in this study were different from the residues at corresponding positions in JFH1 genome. Common mutations identified from different culture-adapted genomes strongly suggest that they play important roles, and likely share common mechanisms, in aiding HCV to break through the restriction of host cells. Understanding the functional roles of these adaptive mutations may open new avenues for the development of culture models for HCV, thus these findings warrant future studies with a focus on the mode of action of adaptive mutations. Additionally, common mutations or mutations occurred at approximate positions frequently identified in genotype 6a or other recombinants, such as amino acids at 348–349 and 2413–2417 (H77 positions), may indicate the importance of corresponding regions in culture adaptation.

To investigate the role of newly identified adaptive mutations in various steps of the CH6acc life cycle, we mutated individually the selected 12 mutations back to the original sequence and tested the viability of resulting mutants in Huh7.5 cells and S29 cells. The S29 cells only support HCV RNA replication, virus particle assembly, and release but not infection, since the Huh7-derived S29 cells were deficient for HCV entry receptor CD81, thus it is suitable for a single-cycle production assay (Russell et al., 2008). In these experiments, we found that CH6acc without L881F or T1285A was severely attenuated and only showed a very low level of replication, without virus spread and detectable infectivity titers in the transfected Huh7.5 cells (Figure 5). Together with the results from single-cycle production assay, in which both -L881F and -T1285A viruses did not produce detectable extracellular infectivity titers and showed only lower intracellular infectivity and RNA titers, we conclude that both L881F and T1285A were critical for HCV RNA replication, virus release, and spread (Figures 5, 6). Given little differences between mutant viruses and CH6acc in intracellular and extracellular infectivity titers, as well as in RNA levels (Figures 5, 6), we conclude that other mutations, including I355M, I413T, Q1120K, F2358C, and E2392G, affected the viability of CH6acc through likely regulating virus assembly, release, and spread.

It is known that the 5′UTR and 3′UTR (UTRs) are essential for HCV RNA replication, translation, and other steps of the HCV life cycle (Honda et al., 1999; Friebe and Bartenschlager, 2002; Friebe et al., 2005; Li et al., 2012a). In the development of infectious culture systems, we also found that the 5′UTR and 3′UTR are very important for the viability of a specific full-length recombinant. The 26 mutations could promote the efficient replication of CH6aORF_26m, which contained the 5′UTR and 3′UTR from JFH1. However, when JFH1 5′UTR and 3′UTR were replaced, respectively, with CH6a-specific 5′UTR and 3′UTR, the viability of virus was slightly attenuated (Figures 4D,E). In CH6a full-length recombinant, nucleotides 1–59 and 9571–9641 were taken from the HK6a and H77, respectively. Several long-range RNA-RNA interactions have been identified, including interactions between two UTRs (5′UTR–3′UTR) (Fricke et al., 2015), both UTRs with NS5B (5′UTR–NS5B-3′UTR) (Diviney et al., 2008; Romero-Lopez and Berzal-Herranz, 2009, 2012; Romero-Lopez et al., 2012), and 5′UTR domain I with Core coding sequences (5′UTR-Core) (Kim et al., 2003). Therefore, the attenuation effect of CH6a 5′UTR and 3′UTR (CH6acc) indicate that such interactions might have been disrupted by the nucleotides differing from JFH1, or CH6a 5′UTR and 3′UTR contain nucleotides unfavorable for the infectivity of virus. Recently, we identified the nucleotides 1, 4, and 35 of genotype 1b strain Con1 were disadvantageous for the infectivity of Con1 5′UTR-NS5A recombinants (Li et al., 2018). However, we could not exclude the possibility that the 5′UTR and 3′UTR of CH6a were involved in other important interactions that are currently unknown, and such interactions were disrupted in CH6aFL_26m virus. This possibility is most likely, because the known interactions exist between the sequences that are conserved across genotypes (Kim et al., 2003; Diviney et al., 2008; Romero-Lopez and Berzal-Herranz, 2009, 2012; Romero-Lopez et al., 2012; Fricke et al., 2015) and all those known interaction-forming sequences in the CH6acc were identical to that in JFH1 and other genotypes. Unlike CH6a UTRs, Pham et al. (2018) recently found that the HK6a UTRs were more efficient than JFH1 UTRs in promoting the virus production of adapted HK6aORF virus. Previously, genotype 2b clone DH10cc also used the UTRs from the other genotype 2b strain J8 (Ramirez et al., 2014). Given the increasing number of viable recombinants that contain different UTRs, we may be expecting to uncover new interactions or functional roles of the 5′UTR and 3′UTR in the complete HCV life cycle.

As a feasible application of infectious culture system, we examined the viral sensitivity to the selected DAAs targeting NS3/4A, NS5A, and NS5B. As expected, CH6a virus CH6aORF_26m and other infectious full-length viruses of genotypes 1 and 2 were inhibited dose-dependently by all DAAs (Figure 8). However, differences in sensitivity between genotype viruses were relatively small (∼10-fold or less) for simeprevir, velpatasvir, and sofosbuvir. For NS5A DAAs, daclatasvir and ledipasvir showed more genotype-dependent sensitivity. For these two inhibitors, greater variations between genotype viruses were also described in a recent report (Gottwein et al., 2018), a comprehensive study for NS5A inhibitors against various genotype viruses. Further, our results and other observations using infectious culture systems showed good correlation with clinical data and clinical trials regarding the efficacy of the tested inhibitors against different genotypes (Gottwein et al., 2011b, 2013; Scheel et al., 2011; Li Y.P. et al., 2014; Voaklander and Jacobson, 2017). Thus, these data also show a clinical relevance of the infectious CH6acc clone and its application in the antiviral study, as well as other basic research.

Genotype 6 viruses are primarily prevalent in Asia, where 60% of the world’s current populations inhabit. Genotype 6 has the greatest genetic diversity (Zhang et al., 2017), of which 6a takes 6.41% of total HCV infections in China and more infections have been reported in some Asian countries (Hubschen et al., 2011; Gower et al., 2014; Li et al., 2015a; Chen et al., 2017). Increasing incidences of genotype 6 are reported in recent years (Chen et al., 2017), and some studies identify that genotype 6 is associated with the high viral load of averagely 5.55 × 10^6^ IU/ml (Chen et al., 2017) and the increasing risk of developing liver cancer (Lee et al., 2017). Thus, development of genotype 6a cell culture system for a genotype 6a isolate meets the critical needs of basic research and clinical applications.

In summary, we have developed a robust culture system for HCV genotype 6a, a genotype particularly prevalent in Asian countries. Infectious culture system represents different genotype isolates will permit genotype- and isolate-specific studies on virus–host interactions. The strategy of using consensus-like clones to construct full-length HCV recombinant may facilitate the culture development of other HCV isolates. Adaptive mutations commonly identified in different recombinants may pave a new path for the development of *in vitro* and *in vivo* infection models for HCV, which will facilitate the studies of other pathogens being difficult to culture.

## Author Contributions

MC and Y-PL designed the research and analyzed the data. MC performed the extensive research and collected the data. FZ, GY, XD, LR, JuL, SF, ZW, MW, YeF, QZ, JiL and KD performed some experiments. CL, JX, GR, YZ, and YoF contributed critical reagents and analytic tools. MC and Y-PL wrote the manuscript.

## Conflict of Interest Statement

The authors declare that the research was conducted in the absence of any commercial or financial relationships that could be construed as a potential conflict of interest.
