# Supplementary material for: Development of an Infectious Cell Culture System for Hepatitis C Virus Genotype 6a Clinical Isolate Using a Novel Strategy and Its Sensitivity to Direct-Acting Antivirals
Source: Front Microbiol. 2018 Dec 4;9:2950. doi: 10.3389/fmicb.2018.02950 (PMC6288186; doi:10.3389/fmicb.2018.02950)
Supplement: Supplementary file 1 [file Presentation_1.pdf]

Development of an infectious cell culture system for hepatitis C virus genotype 6a clinical isolate using a novel strategy and its sensitivity to direct-acting antivirals

Running title: **HCV genotype 6a infectious clone**

Mingxiao Chen[1,2], Fuxiang Zheng[1,2], Guosheng Yuan[3], Xiaobing Duan[1,2], Liang Rong[1,2], Junwei Liu[3], Shengjun Feng[1,2], Ziting Wang[1,2], Min Wang[4], Yetong Feng[1,2], Qing Zhou[1,2], Jinqian Li[1,2], Kai Deng[1,2], Chunna Li[5], Jinyu Xia[5], Guirong Rao[6], Yuanping Zhou[3], Yongshui Fu[4] and Yi-Ping Li[1,5]*

[1]Institute of Human Virology and Zhongshan School of Medicine, Sun Yat-sen University, Guangzhou, China; [2] Key Laboratory of Tropical Disease Control of Ministry of Education, Sun Yat-sen University, Guangzhou, China; [3] Department of Infectious Diseases, Nanfang Hospital, Southern Medical University, Guangzhou, China; [4]Guangzhou Blood Center, Guangzhou, China; [5]Program of Pathobiology, The Fifth Affiliated Hospital and Zhongshan School of Medicine, Sun Yat-sen University, Guangdong, China; [6]Key Laboratory of Liver Disease, Center of Infectious Diseases, PLA 458 Hospital, Guangzhou, China.

*Correspondence author
E-mail: lyiping@mail.sysu.edu.cn (Y.-P.L.)

Supplemental Materials

Supplementary Table 1. The primers used for RT-PCRs for CH6a genome from the patient serum and the cultured cells.

Primer	Sequence (5'-3')	Use of the primer
**For patient serum**		
6a-F59	TCCCCTGTGAGGAACTACTGT	PCR fragment 1
6a-R3590	GCCCCATGATAAACAGTCCACA	PCR fragment 1
6a-F3066	GCCCCTCTATACATCTTGCAG	PCR fragment 2
6a-R5893	AGCACATCAACCAACACCCT	PCR fragment 2
6a-F5511	CTAGGCACATCCCCTACCTC	PCR fragment 3
6a-R7943	TAGCATGGCTTCTAACGTCCT	PCR fragment 3
6a-F7603	TCAGGTTCTTGGTCCACTGTGAGCG	PCR fragment 4 (NS5B)
6a-R9404	TGTTGCTAGGGCCGCTCG	PCR fragment 4 (NS5B)
6a-F8981	GACATCTACGGAGTGACCTA	3'UTR
3UTR- R9571	CGGACCTTTCACAGCTAGC	3'UTR
6a-R9413	AAAGGAACGATGGAGTGTTGCTAG	ORF RT
3UTR-R9619	ATGATCTGCAGAGAGACCAGT	3'UTR RT
**For cultured virus**		
**C-NS2 virus**		
JF40	CGACACTCCGCCATGAATCACT	Outer primer of nested PCR
JF62	CCCCTGTGAGGAACTACTGTCT	Inner primer of nested PCR
JR3790	AATGCTCCCCGCTTGTCCCCY	Inner primer of nested PCR
JR3819	TCAAGGTCGAAATGGGTCTC	Outer primer of nested PCR
**C-5A/ORF/FL virus**[a]		
JF40	CGACACTCCGCCATGAATCACT	PCR fragment 1 (outer primer)
JF62	CCCCTGTGAGGAACTACTGTCT	PCR fragment 1 (inner primer)
6a-R5028	TCAAGCCAGTAAACACGCCTTCCC	PCR fragment 1 (inner primer)
6a-R5165	TGAGACACTTCCACATCGTATCCCA	PCR fragment 1 (outer primer)
6a-F4619	CCGTCGCATTCTACAGAGGTGTCG	PCR fragment 2 (outer primer)
6a-F4819	CGGTATCACGGAGCCAACGACGA	PCR fragment 2 (inner primer)
JR8178	AGGAAGCTCCCATTACCGCCTGA	PCR fragment 2 (inner primer)
JR8511	CCGCTAGGGCTTTCACATAGCAT	PCR fragment 2 (outer primer)
JR9398	GTACCTAGTGTGTGCCGCTCTA	ORF PCR fragment 2 (inner primer)
JR9402	GTGTACCTAGTGTGTGCCGC	ORF PCR fragment 2 (outer primer)
JR9405	CTATGGAGTGTACCTAGTGTGTGC	RT

[a]primers used for amplification of full-length CH6a virus are the same as those used for patient serum.

Supplementary Table 2. Different genome organizations of 5’UTR-NS5A, Core-NS5A, or other recombinants with less CH6a sequence tested in this study.

CH6a recombinant	Mutations	Transfections (day)	Results
**Core-NS5A**			
C-5A_WT	None	3 transfections (28)	Negative
C-5A_LS	F1469L/A1677S (LS mutations)	3 transfections (28)	Negative
C-5A_7m	K1303R/F1469L/V1555L/A1677S/K1696R/I1720F/L1795M	3 transfections (42)	Positive, 1-2% in the first week, no spread
C-5A_9m/G	K1303R/F1469L/V1555L/A1677S/K1696R/I1720F/L1795M/S2209I/S2223P/D2987G	3 transfections (28)	Positive, ≤~1‰, no spread
C-5A_7m/G	K1303R/F1469L/V1555L/A1677S/K1696R/I1720F/L1795M/D2987G	3 transfections (35)	1-2% in the first week, no better than 5’UTR-3
C-5A_8m	K1303R/F1469L/V1555L/V1668A/A1677S/K1696R/I1720F/L1795M	3 transfections (28)	Positive, ≤~1‰, no spread
C-5A_10m/G	K1303R/F1469L/V1555L/V1668A/A1677S/K1696R/I1720F/L1795M/S2209I/S2223P/D2987G	3 transfections (41)	Positive, ≤~1‰, no spread
C-5A_16m	I348M/T386I/L777S/S877P/R932G/K1303R/F1469L/V1555L/V1668A/A1677S/K1696R/I1720F/L1795M/ S2209I/S2223P/D2987G	4 transfections (35)	Positive, ≤~1‰, no spread
C-5A_13m	I348M/T386I/L777S/S877P/R932G/K1303R/F1469L/V1555L/V1668A/A1677S/K1696R/I1720F/L1795M	3 transfections (42)	Positive, ≤~1‰, no spread
C-5A_10m	K1303R/F1469L/V1555L/V1668A/A1677S/K1696R/I1720F/L1795M/S2209I/S2223P	3 transfections (28)	Positive, ≤~1‰, no spread
C-5A_11m	I355M/N416S/I831V/L881F/K1303R/F1469L/V1555L/A1677S/K1696R/I1720F/L1795M	3 transfections (28)	
C-5A_15m	F349S/I355M/N416S/V627A/I746M/I831V/L881F/T1285A/K1303R/F1469L/V1555L/A1677S/K1696R/I1 720F/L1795M (C-5A_15m in the text)	2 transfections (14)	Positive, peak at day 13
C-5A_18m	I355M/N416S/I831V/L881F/T1285A/K1303R/F1469L/V1555L/A1677S/K1696R/I1720F/L1795M/F2358C /D2388A/E2392G/D2423G (C-5A_18m in the text)	3 transfections (14)	Positive, peak at day 9
5’UTR-NS5A			
5-5A_WT	None	3 transfections (21)	Negative
5-5A_LS	F1469L/A1677S (LS mutations)	3 transfections (21)	Negative
5-5A_7m	K1303R/F1469L/V1555L/A1677S/K1696R/I1720F/L1795M	3 transfections (35)	~1% in the first week, no spread
5-5A_16m	I348M/T386I/L777S/S877P/R932G/K1303R/F1469L/V1555L/V1668A/A1677S/K1696R/I1720F/L1795M/ S2209I/S2223P/D2987G	3 transfections (42)	~1‰ for 2 weeks, no spread
**CH6a-HK6a-JFH1 chimeras**			
C-NS3_3m*	K1303R/F1469L/V1555L	3 transfections (35)	~1% in the first week, no spread
C-NS2#	None (refers to C-NS2 in the text)	1 transfection (13)	1-5% in the first week, peak at day 25
5’UTR-NS2/NS4A -5A$	None	3 transfections (41)	≤1‰ for 2 weeks, no spread

Mutations containing in different genome organizations were indicated.

*, the remaining genome sequences are JFH1(5’UTR), JFH1(NS4A-3’UTR, with EGFP in NS5A).

#, the remaining genome sequences are J6(5’UTR), JFH1(NS3-3’UTR, with EGFP in NS5A).

$, the remaining genome sequences are JFH1(NS3, NS5B-3’UTR).

Supplementary Figure 1, part I

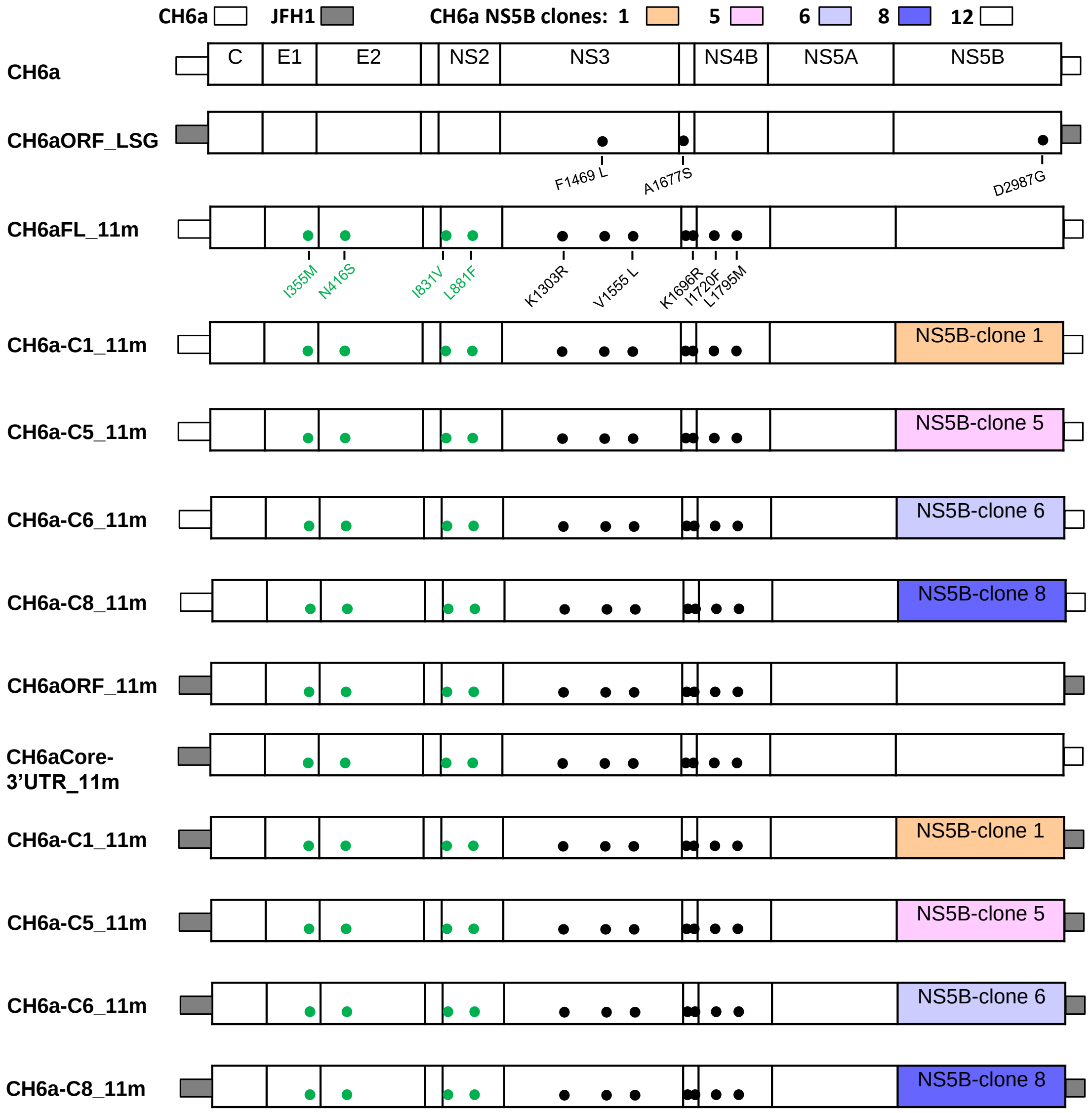

Supplementary Figure 1, part II

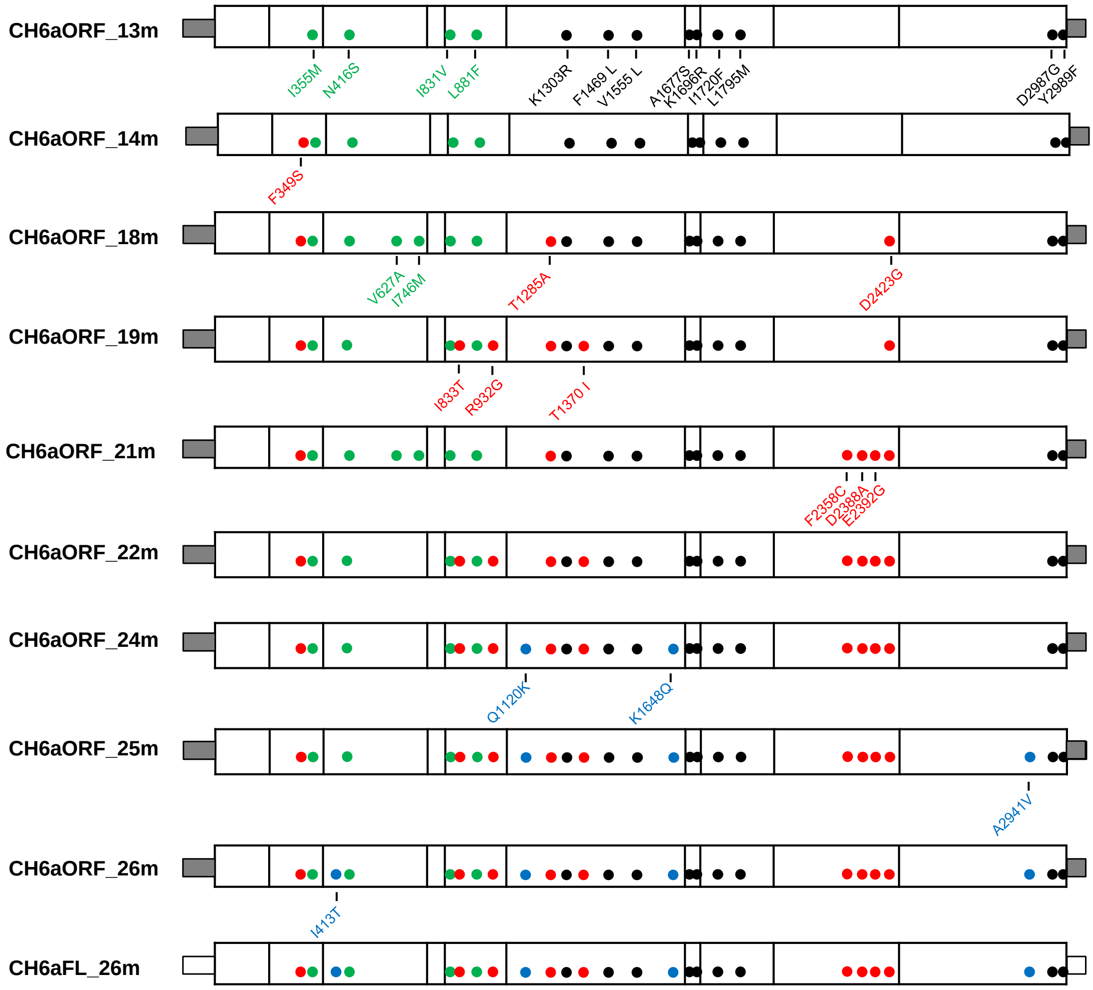

Supplementary Figure 1. CH6a ORF or full-length genomes with mutations tested in this study. Schematic diagrams of CH6a ORF recombinant with 5'UTR and 3'UTR from JFH1 or full-length genomes with NS5B sequences derived from different clones of PCR products tested in this study. Combinations of mutations engineered into the genomes were indicated. Mutations were highlighted in color, in which mutations identified previously were in black color, newly identified from the Core-NS2 recombinant were in green color (see Table 1), from the passaged C-5A_11m viruses were red color (Table 2), and from CH6aORF_22 were in blue color (Table 3).
